# Utilizing Machine Learning for Predicting PrEP Use Status Among Sexual and Gender Minority Young Adults

**DOI:** 10.1007/s11121-025-01872-1

**Published:** 2026-01-10

**Authors:** Callisto Boka, Elizabeth A. Yonko, Mehrab Beikzadeh, Kimmo Kärkkäinen, Chenglin Hong, Majid Sarrafzadeh, Ian W. Holloway

**Affiliations:** 1 Department of Epidemiology, University of California, Los Angeles, CA USA; 2 Department of Computer Science, University of California, Los Angeles, Los Angeles USA; 3 Optum, Los Angeles USA; 4 School of Social Work, University of Connecticut, Hartford, CA USA; 5 School of Nursing, University of California, Los Angeles, Los Angeles USA

**Keywords:** Sexual and gender minorities, Young adults, Pre-exposure prophylaxis (PrEP), MHealth, Machine learning model

## Abstract

Pre-exposure prophylaxis (PrEP) is a highly effective biomedical prevention tool for HIV yet remains underutilized among key populations, particularly among young sexual and gender minorities (SGM). Recognizing the popularity of specific dating and social media apps among SGM young adults, we leveraged user data from these platforms to build a machine learning (ML) model that could inform targeted, data-driven interventions aimed at improving PrEP uptake and adherence. We adapted eWellness, an Android mobile app, to passively collect data from research participants capturing mobile app usage, keystroke patterns and logs, and GPS location data between 2021 and 2024. These data were used to train a ML model to predict self-reported PrEP use. Model accuracy was evaluated through F1 scores across different data types and feature combinations. Study protocols were developed in collaboration with community partners and adhered to strict ethical and privacy standards. A total of 82 SGM young adults participated, with 46 (56%) reporting PrEP use at baseline. Our machine learning model demonstrated good predictive accuracy for predicting PrEP use and non-use, achieving an F1 score of 0.84 (PrEP use) and 0.82 (non-use) outcomes when incorporating data from all mobile apps, including messaging, dating, and social media mobile apps. By contrast, predictions based solely on social media mobile app usage, language associated with sexual behavior and substance use risk, or location monitoring demonstrated worse accuracy (F1 scores of 0.79/0.75, 0.70/0.57, and 0.70/0.52, respectively). Additional feature extraction methods, as well as various combinations of these features, were also tested. However, none achieved predictive accuracy as well as the model incorporating all mobile app usage data combined. This study demonstrates the potential of machine learning to accurately predict PrEP use status among SGM young adults. The findings offer a foundation for developing more personalized PrEP promotion strategies, particularly among SGM young adults who use social media and dating apps. Future research should assess the model’s adaptability across diverse SGM subgroups to further inform intervention development. Registry: ClinicalTrials.gov, ID: NCT04710901, November 9, 2020.

## Introduction

The United States (US) has made significant strides in the last decade towards ending the HIV epidemic (EHE), largely due to pre-exposure prophylaxis (PrEP), a highly effective biomedical prevention tool. However, disparities in PrEP uptake persist among key populations at risk for HIV, particularly among Black/African American and Hispanic/Latino men who have sex with men (MSM), transgender women, and youth (Harawa et al., 2022; Huang et al., 2018; Kanny et al., 2019; Snowden et al., 2017; Sullivan et al., 2020, 2024). Despite accounting for nearly one-fifth of new HIV cases, youth aged 13 to 24, many of whom are sexual and gender minorities (SGM), comprise only 12% of PrEP users in the US (Sullivan et al., 2020).

Substance use further contributes to HIV risk by both limiting PrEP access and increasing sexual risk behaviors (Shuper et al., 2020). SGM experience higher rates of substance use compared to their heterosexual and cisgender peers, which has been linked to minority stress, mental health comorbidities, and “chemsex”/sexualized drug use (SDU) culture (Cochran et al., 2002; Demant et al., 2016; Green & Feinstein, 2012; Hughes & Eliason, 2002; Marques Oliveira et al., 2023; Maxwell et al., 2019; Owens & Montemayor, 2025; Parent et al., 2019; Tomkins et al., 2019). Prevalence estimates for sexualized drug use (SDU) among SGM range from 10 to 94% (Jalil et al., 2022; Marques Oliveira et al., 2023; Maxwell et al., 2019; Owens & Montemayor, 2025; Tomkins et al., 2019), and prior research has found SDU to be associated with condomless anal sex and increased numbers of sexual partners, increasing risk for HIV and STI acquisition (Colfax & Shoptaw, 2005; Edmundson et al., 2018; Halkitis et al., 2009; Luk et al., 2016; Maxwell et al., 2019; Rivera et al., 2021; Strathdee et al., 2021; Tomkins et al., 2019).

These behavioral and structural disparities are deeply intertwined with digital environments, as young SGM increasingly use dating apps and social media to find both sex and substance use partners (Holloway et al., 2014, 2024). These same platforms could also be a source of rich, real-time data to understand how these digital environments shape HIV risk. These methods align with earlier implementation science reviews showing the unique value of internet-mediated HIV prevention research with MSM populations (Grov et al., 2019). Prior mHealth interventions have leveraged mobile phones to deliver reminders, education, and counseling (Belzer et al., 2025; Biello et al., 2023, 2024; Chan et al., 2021; Mimiaga et al., 2017, 2018; Mustanski et al., 2023; Parsons et al., 2019; Swendeman et al., 2024), but most relied on self-report measures prone to recall bias and potentially burdensome to participants.

Advances in mobile sensing and machine learning (ML) offer new opportunities to move beyond self-report, by passively capturing behavioral signals and identifying patterns linked to PrEP use. Prior research has found that the ML approach to analyzing social media and online data has important applications in public health such as for predicting health-related outcomes (Ali et al., 2021; Li et al., 2020; Lopez-Castroman et al., 2020; Velasco et al., 2014; Włodarczyk et al., 2021). In our prior work, for example, we predicted methamphetamine use, injection drug use, and sexual risk behaviors (i.e., condomless anal sex, having more than six sexual partners, and having a sexual partner living with HIV) with a good level of accuracy (Beikzadeh et al., 2025). This approach is guided by the Information-Motivation-Behavioral Skills (IMB) model, which posits that accurate HIV prevention information, motivation to engage in protective behaviors, and the behavioral skills to act on that motivation together determine preventive practices such as PrEP uptake (Fisher & Fisher, 1992; Fisher et al., 2003). Digital platforms, particularly dating and social networking apps, may influence both motivation and behavioral skills by facilitating sexual partner-seeking, communication, and community norms, making them important contexts for understanding and predicting PrEP use.

To our knowledge, this ML approach to identifying patterns in mobile sensing data has not yet been used to predict protective behaviors for HIV prevention, such as PrEP use, which could have benefits for delivering “just-in-time” interventions for young SGM individuals at risk for HIV. As such, in this study, we developed an Android mobile sensing application (eWellness) which tracks location, keystroke data, and participant app use. We then trained ML models to detect PrEP use status from these data and evaluated their performance.

The main contributions of this study are the following:

1. Development and validation of a machine learning algorithm to predict PrEP use among SGM young adults
2. Evaluation of the types of data (i.e., social media, location, risk-indicating word use) that should be collected to identify differences in PrEP use behaviors among SGM young adults using a community-centered approach
3. Measuring the accuracy of our machine learning algorithm to predict PrEP use, and discussion of implications for future use as a preventative tool in HIV acquisition

## Methods

### Data Source

The present research sources data from the uTECH Study, a randomized controlled trial (ClinicalTrials.gov ID: NCT04710901) (Holloway, 2024) taking place between November 2020 and June 2024. The study aimed to measure the acceptability, appropriateness, and feasibility of a novel SMS-based HIV intervention, “uTECH,” over a 12-month period. Participation included a baseline assessment, periodic interviews, survey assessments, and online counseling sessions.

The detailed research protocol for the uTECH Study has been previously published and contains additional information on original aims, design, eligibility, screening, recruitment, and data collection (Holloway et al., 2024).

### Eligibility and Recruitment

Participants were eligible if they met the following criteria at baseline: (1) aged 18 to 29 years; (2) identified as a transgender man or woman, cisgender man, or non-binary person; (3) engaged in anal or oral sex with a man in the past 3 months; (4) used substances such as alcohol, marijuana, poppers (amyl nitrate), methamphetamines, heroin, cocaine, or ecstasy in the past 3 months; (5) had sex while using substances in the past 3 months; (6) were HIV-negative or had an unknown HIV status; (7) used a dating app to meet sexual or substance use partners in the past 3 months; (8) owned a smartphone; (9) resided in the US; (10) agreed to participate in a 12-month study; and (11) provided informed consent. Participation was entirely remote.

Recruitment of participants and baseline interviews took place between October 2021 and May 2023. Participants were predominantly recruited by digital advertisements on sites such as Grindr, Instagram, Facebook, Reddit, and X. These advertisements linked to a screener that was verified and checked for fraud by the research team before scheduling a baseline interview via videoconferencing platform, Zoom. During the baseline interview, participants installed the eWellness app (see “eWellness Data Collection App” below) with a member of the research team. Upon installation of the app, participants completed a brief quantitative survey on sociodemographics and past 3-month substance use and sexual behavior.

### Sample Size

A total of 423 participants were recruited and completed a baseline assessment, of whom 103 (24%) were Android users and consented to downloading the data collection app, “eWellness.” The present research limits the sample to Android users. Missing data was handled with complete-case analysis. Only Android users who had 30 days or more of data collected over 12 months were included as our analytic sample, totaling 82 participants. To evaluate potential bias from missing data, we compared participants included in the analytic sample with those excluded on key sociodemographic and behavioral variables (e.g., age, gender identity, race, Hispanic/Latino ethnicity, sexual orientation, education, PrEP use, number of sexual partners, recent receptive condomless anal sex, recent methamphetamine use, and recent injection drug use) using chi-square tests of independence at a significance level of *p* < 0.05.

To determine the “true” PrEP status of the sample for validation of the model prediction, all participants were asked during the baseline survey whether they were currently using PrEP. Participants who reported current oral PrEP use were asked to upload a photo of their PrEP prescription or pill bottle, and study staff followed up as needed until documentation was received. Of 82 participants, just over half (*n* = 46, 56%) self-reported active PrEP use at baseline, and all but one provided acceptable photo verification. The single exception was a participant using long-acting injectable PrEP, whose status was confirmed through repeated self-report.

### eWellness Data Collection App

Participants who used Android smartphones, met the eligibility criteria, consented to participate, and enrolled in the study were instructed to install the study’s data collection app, “eWellness,” on their primary devices. Due to the incompatibility of the app with iOS-based devices, this app was not available for participants who used iPhone devices.

The app utilized the Android framework AWARE (Ferreira et al., 2015; Van Berkel et al., 2023) to passively collect the following information: keyboard input, location, and which specific app or website (via mobile browser) that keyboard input was being typed into. During the consent process, participants were informed in detail of the types of data being collected and precautions taken to ensure data privacy, confidentiality, and compliance with ethical guidelines. Upon either discontinuing the study by request or at offboarding, research staff would instruct the participant to remove the app from their phone, ceasing all data collection. Greater detail on server information and data preprocessing from the eWellness app has been previously published (Beikzadeh et al., 2025).

### Feature Extraction

Feature extraction and data preprocessing in the present research follow the approach detailed in Beikzadeh et al. (2025). To summarize, after data cleaning, we extracted behavioral, linguistic, and mobility-related features from social media, messaging (such as Facebook Messenger, WhatsApp, SMS texting, etc.), and location data collected by the eWellness app during participation.

### Text Data

We analyzed keyboard data inputted by participants while using social media, dating, and messaging apps. Text data underwent lemmatization using the WordNet Lemmatizer from the Natural Language Toolkit (NLTK) (Bird et al., 2009) to standardize word forms. Stop words, placeholder text, and infrequent words (used by fewer than five participants) were removed. Word frequencies were computed to quantify language use.

Sentiment embeddings were generated using a pre-trained Bidirectional Encoder Representations from Transformers (BERT) language model (Devlin et al., 2019), which was fine-tuned on social media text for optimal contextual understanding.

### Risk-Indicating Words

We extracted features including words indicating behavior associated with increased risk of HIV seroconversion and substance use, referred to as “risk-indicating words,” which began from a pre-defined phrase list validated in prior research (Ovalle et al., 2021), to then be refined for this research population. Semi-structured interviews with participants were conducted in an earlier phase of the project to gather and assess existing terms and emojis associated with sex and/or drug use (ex., “orgy,” “crystal,” “party-n-play”). Participants would provide insight into their interpretations of various terms in the word library, and terms would be removed or added based on feedback to ensure accuracy to population-specific meanings and promote direct community involvement.

### App Data

We computed frequency-based metrics (days using specific app out of days using any apps) to measure app engagement, focusing on social, messaging, and dating apps while filtering out non-relevant categories such as music and shopping apps.

### Location Data

We applied the Mean-Shift clustering algorithm (Cheng, 1995) to GPS coordinates to identify frequently visited locations, assuming the most visited cluster represented a participant’s home. From these clusters, we extracted mobility-based features such as daily unique locations, total distance traveled from home, and overall mobility range. To safeguard confidentiality, raw GPS coordinates were never stored. We applied Mean-Shift clustering to group nearby points and retained only cluster centroids to derive mobility features (e.g., daily unique locations, distance traveled) (Cheng, 1995).

### Linguistic Inquiry and Word Count (LIWC)

LIWC is a validated text analysis tool that matches each word to psychological dictionaries and reports the percentage of words in each category (Pennebaker et al., 2015; Tausczik & Pennebaker, 2010). For example, if 50 of 1000 words reflect positive emotion, the score is 5%. Dictionaries capture constructs such as cognitive processes and social affiliation (Boyd et al., 2022). Scores were aggregated across all participant text for modeling. This approach allowed us to explore whether language patterns reflecting cognitive processing, social engagement, and affective tone differed between PrEP users and non-users.

### Model Training

We trained logistic regression (Cox, 1958) and gradient boosting (Friedman, 2001) models using Scikit-learn (Pedregosa et al., 2012) to predict survey responses of either PrEP use or PrEP non-use outcomes, employing leave-one-out cross-validation to account for the small sample size. Five feature categories derived from passively collected smartphone data were entered into the models: app usage metrics (frequency of social, messaging, and dating app use), text-based features (BERT embeddings and counts and categories of risk-indicating words), location features (mobility metrics from Mean-Shift–clustered GPS data), LIWC linguistic categories (cognitive, social, affective), and selected baseline demographic variables.

Feature inclusion followed a domain-driven process informed by behavioral health theory and prior research linking digital behavior to HIV risk and prevention. To manage the high feature-to-sample ratio (*n* = 82), we applied variance-threshold filtering to remove near-zero-variance predictors, standardized all features, and reduced dimensionality with TruncatedSVD (e.g., BERT embeddings from 768 to 50 components; LIWC, location, social-app, and all-app features to 20 components). We then systematically tested individual feature categories and their combinations to identify the most predictive sets. Logistic regression served as the primary model for interpretability, and the gradient boosting model was tuned conservatively to limit overfitting.

### Model Evaluation

**Fig. 1:**
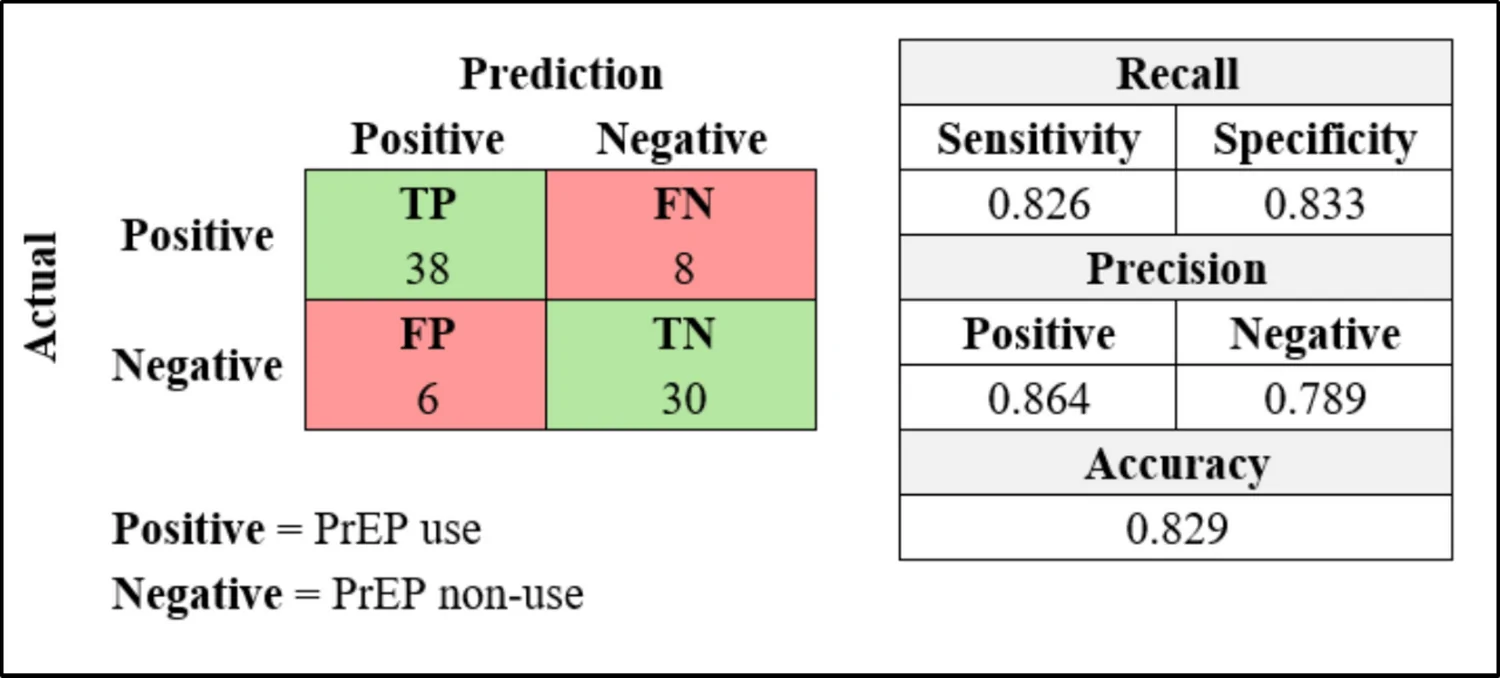
Confusion matrix and performance metrics for a logistic regression model predicting PrEP use. The model achieved sensitivity of 0.826, specificity of 0.833, precision of 0.864 (PrEP use) and 0.789 (non-use), and overall accuracy of 0.829, with 38 true positives, 30 true negatives, 8 false negatives, and 6 false positives

Model performance was evaluated using F1 scores as the primary metric, with precision, recall, sensitivity, specificity, and overall accuracy reported as secondary metrics. We also assessed model discrimination using receiver operating characteristic (ROC) and precision–recall (PR) curves, which are more informative than ROC curves under modest class imbalance (Bradley, 1997; Saito & Rehmsmeier, 2015). Confusion matrix results for the logistic regression model are shown in Figure 1. ROC and PR curves for the best-performing models are presented in Figures 4 and 5, and full model comparisons are summarized in Table 3.

### Model Interpretability

**Fig. 2:**
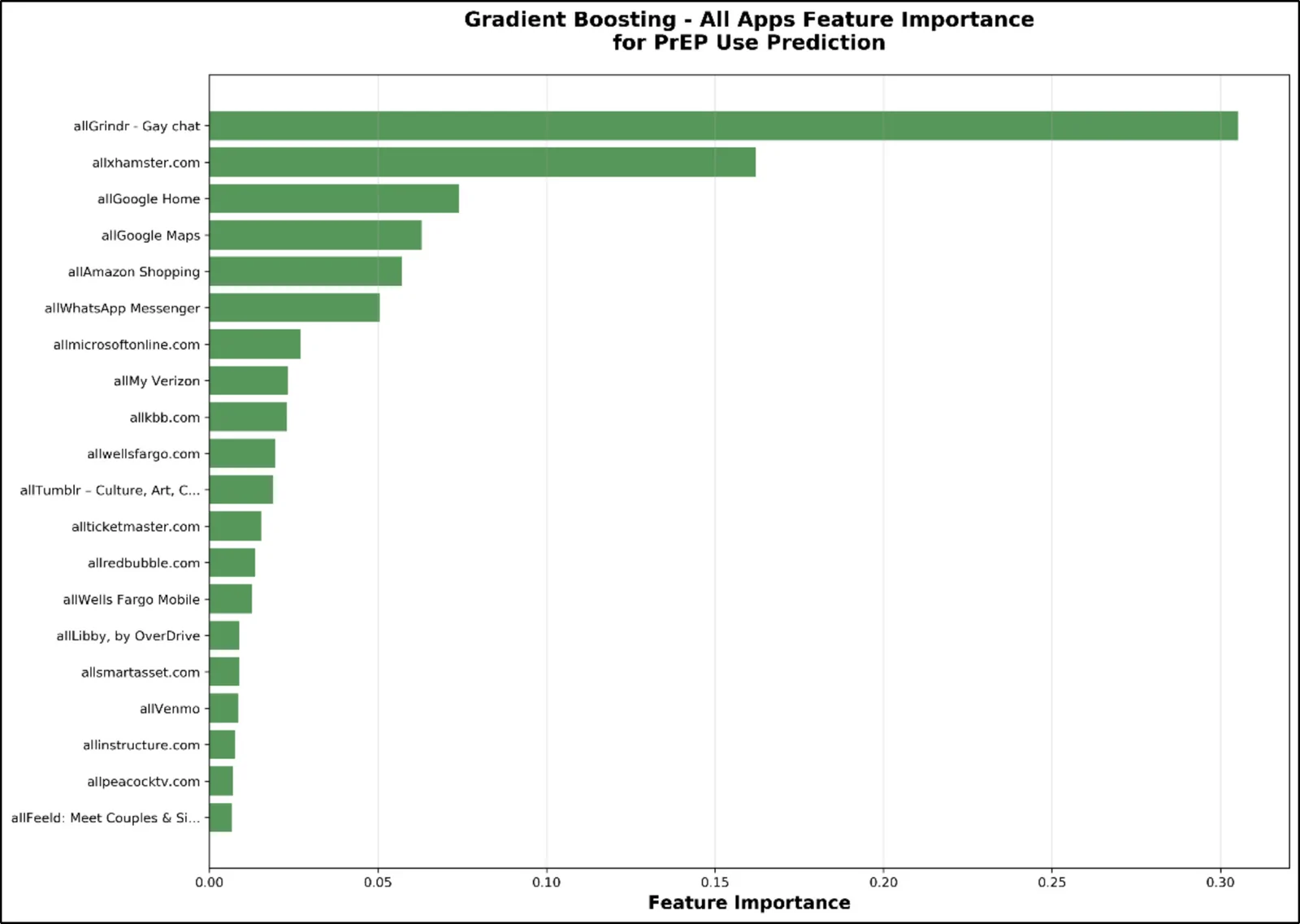
Feature importance from the gradient boosting “All Apps” model predicting PrEP use. The model (F1 = 0.84) identified Grindr usage as the best predictor, followed by xHamster, Google Home/Maps, Amazon Shopping, and WhatsApp Messenger. Importance scores represent each feature’s relative contribution to classification accuracy

**Fig. 3:**
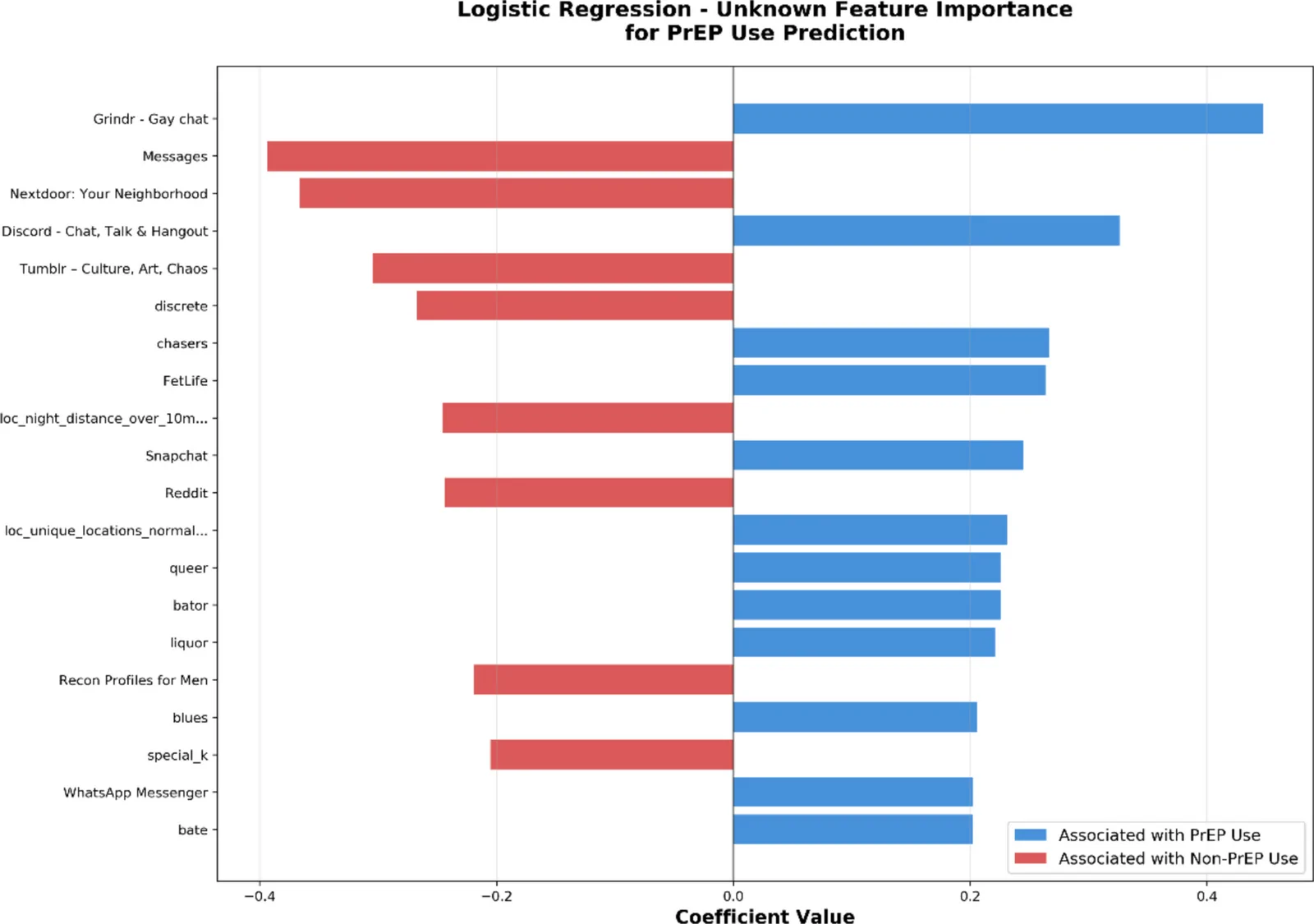
Standardized logistic regression coefficients for PrEP use prediction. Positive values (blue) indicate association with PrEP use and negative values (red) with non-use. Messaging activity, Grindr, and several social or cognitive language features were most strongly linked to PrEP use, while apps such as Nextdoor and Discord showed negative associations

To enhance transparency, we examined model interpretability by analyzing (a) standardized logistic regression coefficients and (b) gradient boosting feature importance scores. Coefficients indicate the direction and magnitude of associations between each feature and PrEP use, while tree-based importance scores quantify each feature’s relative contribution to classification accuracy. These approaches follow best practices for interpretable machine learning analyses in health research (Bradley, 1997; Pennebaker et al., 2015; Tausczik & Pennebaker, 2010). Feature importance plots and coefficient estimates for the top models are shown in Figures 2 and 3.

### Feature Analysis

We measured differences in behavioral patterns between PrEP use and non-use groups by analyzing individual features. For app usage, location, and LIWC features, we first normalized each feature to a 0–1 range by dividing by its maximum value. We then calculated means for each group (PrEP users vs non-users) across feature-specific dimensions. App usage normalized means represent usage rates across app categories (messaging, social media, and dating; see Figure 6). Location means were calculated by the number of locations, proportion of time away from home, and distance from home (see Figure 8). And LIWC feature means reflect usage within linguistic categories, including social processing, cognitive processing, perception, physical, drives, and affect (see Figure 9).

Analysis of risk-indicating word use diverted slightly from this formula; instead, PrEP use and non-use groups were compared using frequency metrics representing how often sex- and drug-related words appeared in text-inputted data. These comparisons relied on mean frequency counts rather than normalized values (see Figure 7). For full methodological details regarding model development, refer to Beikzadeh et al. (2025).

## Results

### Participant Characteristics

Sociodemographic, sexual behavior, and substance use characteristics of the study population are summarized in Table 1. The sample consisted of 82 participants, with 46 (56%) reporting PrEP use and 36 (44%) reporting no PrEP use. The average age of participants was 25.0 years (SD = 2.83).

**Table 1:** Participant characteristics, stratified by PrEP status at baseline

Characteristic	PrEP use		PrEP non-use		Total	
	*N*	%	*N*	%	*N*	%
Participants	46	56%	36	44%	82	100.0%
Age	Mean (SD)		Mean (SD)		Mean (SD)	
	25.7 (2.36)		24.1 (3.15)		25.0 (2.83)	
Race	***N***	**%**	***N***	**%**	***N***	**%**
American Indian or Native Alaskan	1	2.2%	1	2.8%	2	2.4%
Asian	9	19.6%	4	11.1%	13	15.9%
Black or African American	1	2.2%	6	16.7%	7	8.5%
Hispanic or Latino	8	17.4%	2	5.6%	10	12.2%
Middle Eastern and North African	0	0.0%	1	2.8%	1	1.2%
Two or more races	4	8.7%	7	19.4%	11	13.4%
Non-Hispanic White	23	50.0%	15	41.7%	38	46.3%
Gender						
Cisgender man	34	73.9%	20	55.6%	54	65.9%
Transgender man	7	15.2%	7	19.4%	14	17.1%
Non-binary	3	6.5%	8	22.2%	11	13.4%
Transgender woman	2	4.3%	1	2.8%	3	3.7%
Sexual orientation						
Gay	32	69.6%	23	63.9%	55	67.1%
Bisexual or pansexual	9	19.6%	11	30.6%	20	24.4%
Queer	3	6.5%	2	5.6%	5	6.1%
Straight or heterosexual	1	2.2%	0	0.0%	1	1.2%
Refuse to answer	1	2.2%	0	0.0%	1	1.2%
Education						
Less than college degree	14	30.4%	19	52.8%	33	40.2%
College degree or higher	32	69.6%	16	44.4%	48	58.5%
Refuse to answer	0	0.0%	1	2.8%	1	1.2%
US region						
West	15	32.6%	11	30.6%	26	31.7%
Northeast	14	30.4%	11	30.6%	25	30.5%
South	7	15.2%	10	27.8%	17	20.7%
Midwest	10	21.7%	4	11.1%	14	17.1%
Sexual behavior^a^						
Condomless receptive sex	35	76.1%	26	56.5%	61	74.4%
6 or more sexual partners	29	63.0%	12	26.1%	41	50.0%
Substance use^a^						
Methamphetamine use	9	19.6%	6	13.0%	15	18.3%
Injection drug use (IDU)	2	4.3%	1	2.2%	3	3.7%

^a^Within 3 months prior to baseline

Regarding gender identity, most participants identified as cisgender men (65.9%), while 17.1% identified as transgender men, 13.4% identified as non-binary, and 3.7% identified as transgender women. In terms of sexual orientation, the majority identified as gay (67.1%), followed by bisexual or pansexual (24.4%), queer (6.1%), and straight or heterosexual (1.2%).

When comparing PrEP users to non-users, PrEP users were in larger proportion: non-Hispanic White (50.0% vs 41.7%), Asian (19.6% vs 11.1%), Hispanic or Latino (17.4% vs 5.6%), cisgender men (73.9% vs 55.6%), gay (69.6% vs 63.9%), had a college degree or higher (69.6% vs 44.4%), and resided in the Midwest (21.7% vs 11.1%) and West (32.6% vs 30.6%) regions of the US PrEP users in our sample were also slightly older on average (*M* = 25.7, SD = 2.36) than participants who did not use PrEP (*M* = 24.1, SD = 3.15).

To evaluate potential bias from missing data, we compared participants included in the analytic sample with those excluded on key sociodemographic and behavioral variables (age, gender identity, race, Hispanic/Latino ethnicity, sexual orientation, education, PrEP use, number of sexual partners, recent receptive condomless anal sex, recent methamphetamine use, and recent injection drug use) using chi-square tests of independence; only PrEP use differed significantly, with PrEP use being higher among the analytic sample of participants (*χ*^2^ = 4.09, df = 1, *p* = 0.0432).

### Data Collected

Data collection took place between November 2021 and April 2024. Our detailed study protocol has been previously described (Beikzadeh et al., 2025; Holloway et al., 2024). Table 2 summarizes the volume of data collected by type. Passive data collection resulted in obtaining an average of 59,129.7 lines of text data, 16,917.4 locations, and 92.1 unique apps from each participant’s Android device.

**Table 2:** Summary of mobile data collected from participants

	Total	Mean	Median	SD	Min	Max
Lines of text	4,848,639	59,129.7	45,758	43,832.2	2529	195,797
Locations	1,607,149	16,917.4	6071	26,793	509	169,334
Unique apps	2248	92.1	89.5	46.4	20	289

### Model Performance

Our best-performing model was gradient boosting using All Apps (keystroke-derived text from any app), yielding F1 = 0.84 (PrEP use) and 0.82 (non-use) (Table 3). We found the best predictive accuracy in both models for PrEP use and non-use (0.84 and 0.82, respectively) when using keystroke-derived text data collected from any app, including messaging, social media, and dating apps. Overall, our models predicted PrEP use with better accuracy compared to non-use.

**Table 3:** F1 score results for predicting PrEP use by feature or combination of features, comparing simple logistic regression to gradient boosting results

Feature(s)	F1 scores			
	PrEP users		PrEP non-users	
	Logistic regression	Gradient boosting	Logistic regression	Gradient boosting
Social apps	0.62	0.79	0.54	0.75
All apps	0.63	0.84	0.49	0.82
Location	0.67	0.70	0.41	0.52
Risk-indicating words	0.64	0.70	0.58	0.57
All words	0.73	0.53	0.55	0.35
LIWC^a^	0.49	0.61	0.31	0.46
BERT^b^	0.65	0.61	0.54	0.50
Risk-indicating words, LIWC	0.65	0.63	0.52	0.49
Social apps, risk-indicating words	0.66	0.70	0.58	0.60
Social apps, risk-indicating words, LIWC	0.65	0.72	0.56	0.63
Social apps, risk-indicating words, location	0.74	0.78	0.62	0.66
Social apps, risk-indicating words, location, LIWC	0.70	0.74	0.59	0.59
All	0.71	0.72	0.60	0.59

^a^*LIWC*, linguistic inquiry and word count

^b^*BERT*, Bidirectional Encoder Representations from Transformers

The classification matrix of our logistic regression model’s prediction of PrEP use (positive class) and PrEP non-use (negative class; prior to using a gradient boosting classifier) is displayed in Fig. 1. Overall accuracy of the model was 0.829, with a sensitivity of 0.826 and specificity of 0.833. Precision of predicting the positive class was 0.864 and of the negative class 0.789.

### Feature Importance

Figure 2 shows the gradient boosting feature importance plot for the “All Apps” model (F1 = 0.84). Grindr usage was the best predictor, followed by other app engagement metrics (e.g., xHamster, Google Home/Maps, Amazon Shopping, WhatsApp Messenger).

Figure 3 displays standardized logistic regression coefficients, where positive values (blue) indicate features associated with PrEP use and negative values (red) indicate association with non-use. Messaging activity, Grindr, and cognitive/social language features were positively associated with PrEP use.

**Fig. 4:**
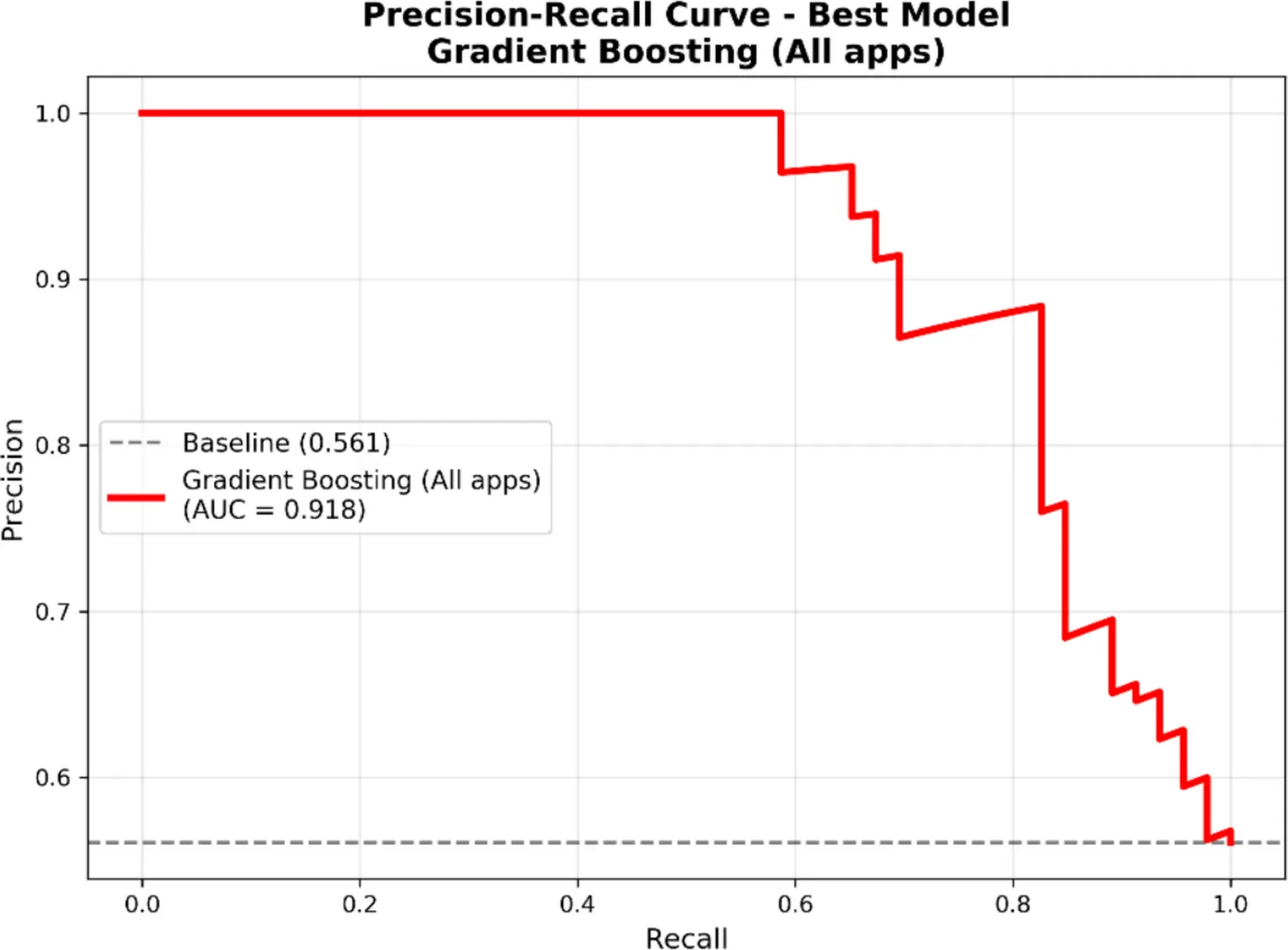
Precision–recall curve for the best gradient boosting “All Apps” model. The model achieved an area under the curve (AUC) of 0.918 compared with a baseline precision of 0.561, demonstrating high precision across a range of recall thresholds despite the modest class imbalance (56% PrEP users)

Figure 4 presents the PR curve for the gradient boosting “All Apps” model. The area under the curve was 0.918, substantially higher than the baseline precision of 0.561, indicating strong precision at varying recall levels even with the unequal distribution of PrEP users and non-users.

**Fig. 5:**
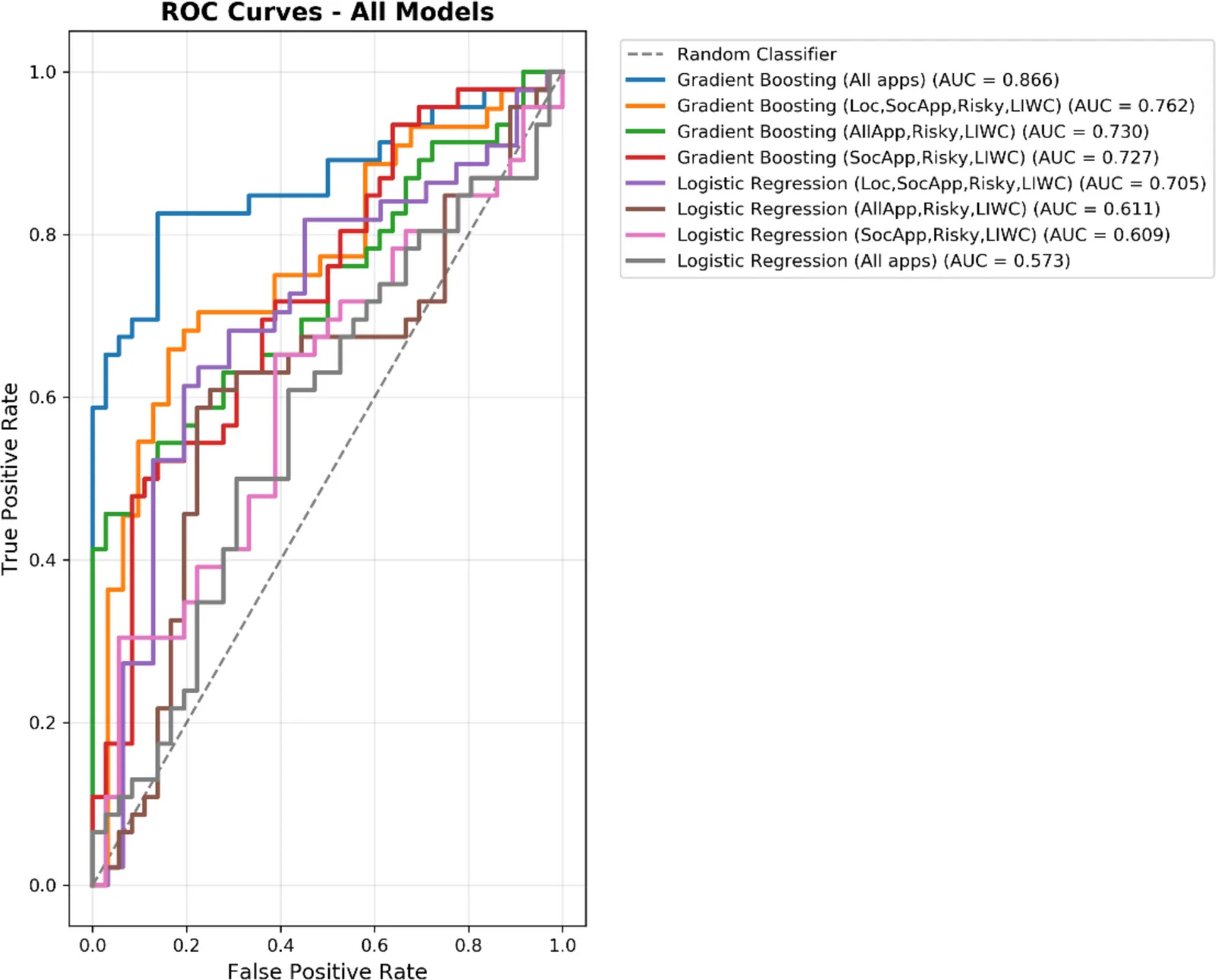
Receiver operating characteristic (ROC) curves comparing all tested models and feature sets. The gradient boosting “All Apps” model achieved the highest discriminative performance (AUC = 0.866), outperforming logistic regression models (best AUC = 0.573) and gradient boosting models limited to specific feature subsets

Figure 5 presents ROC curves comparing all models; the gradient boosting “All Apps” model achieved the highest AUC (0.866), outperforming logistic regression (AUC = 0.573) and all other feature combinations.

### Feature Analysis

Next, we examined differences between PrEP users and non-users within various components of the following features: app use, risk-indicating words, location, and LIWC. This analysis explores how these groups may differ in their use of technology, language, and physical movement patterns.

#### App Use

**Fig. 6:**
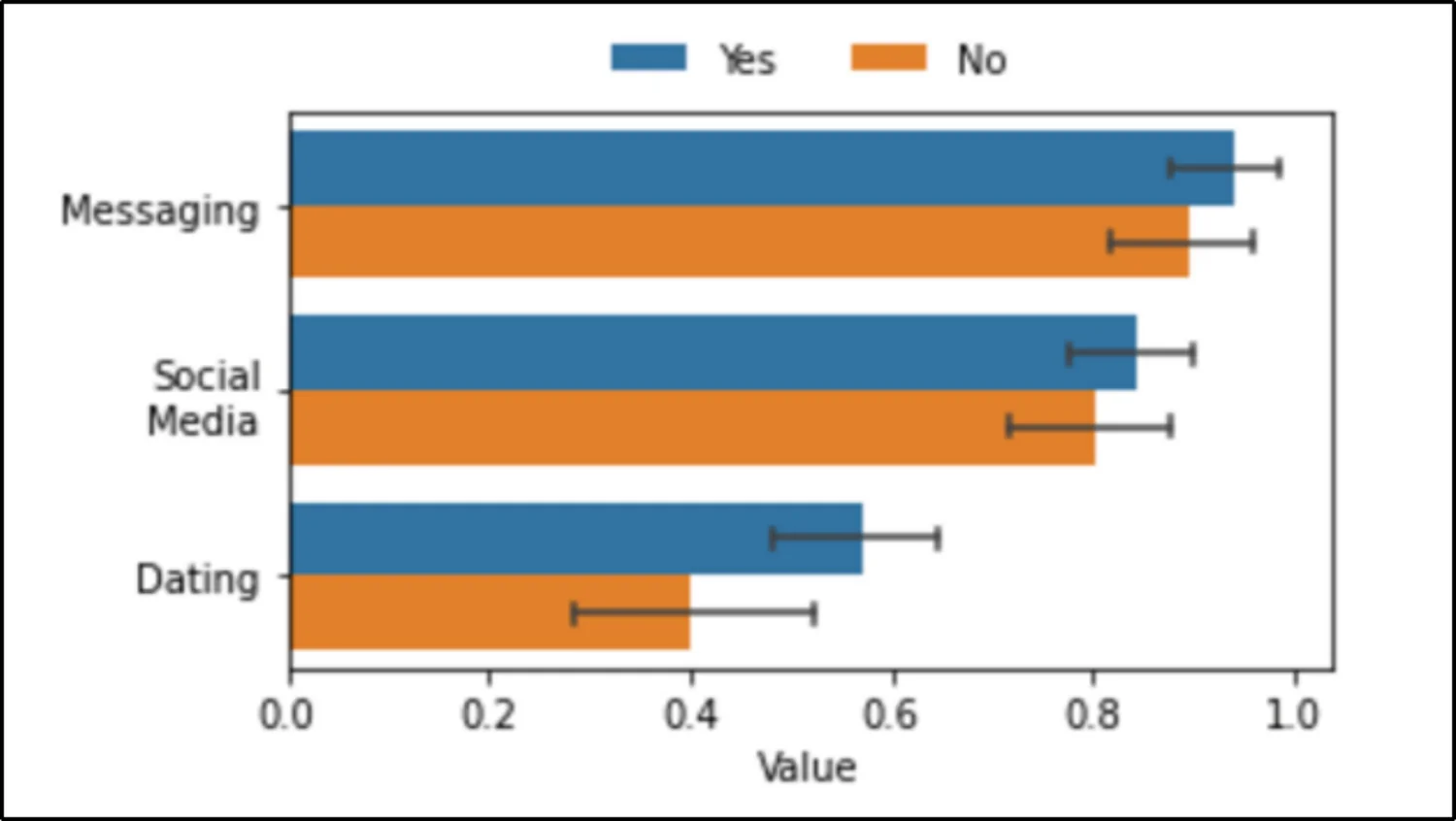
Normalized mean app use among PrEP users and non-users, with error bars indicating standard deviations. PrEP users reported higher average use across messaging, social media, and dating apps compared with non-users

Comparisons of normalized mean app use rates between PrEP users and non-users by category of app are shown in Fig. 6. PrEP users and non-users reported similar levels of app usage across different app categories, with a notable difference in messaging app use. PrEP users were significantly more likely to use messaging apps than non-users (*p* = 0.02), with a mean of 0.57 compared to 0.40. Social media app usage was slightly higher among PrEP users (0.94) compared to non-users (0.89), but this difference was not statistically significant (*p* = 0.33). Similarly, dating app usage showed a small, non-significant difference between PrEP users (0.84) and non-users (0.80, *p* = 0.44).

#### Risk-Indicating Words

**Fig. 7:**
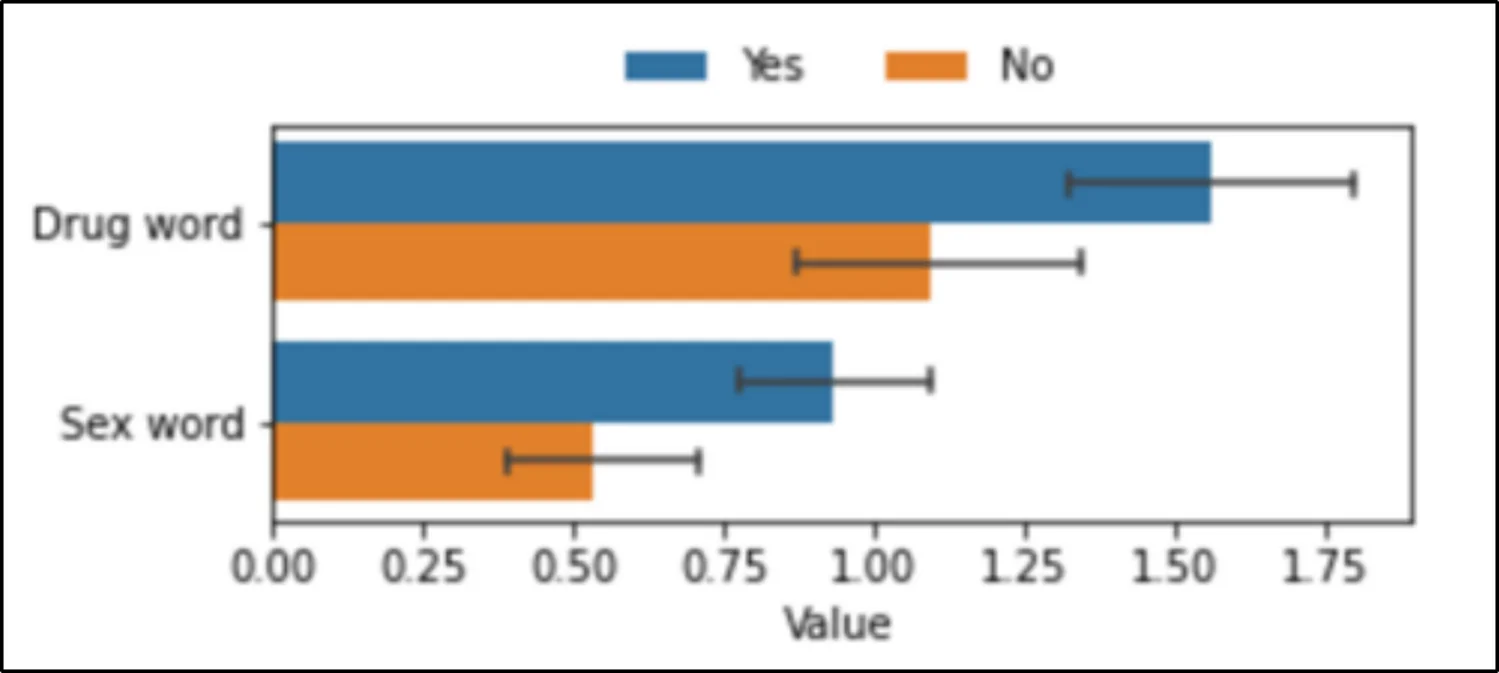
Mean frequency of sex- and drug-related word use among PrEP users and non-users, with error bars representing standard deviations. PrEP users showed higher average usage of both word types compared to non-users

PrEP users were significantly more likely to use both sex- and drug-related words compared to non-PrEP users. As shown in Fig. 7, the non-standardized mean frequency of drug-related words was higher among PrEP users (1.56) than among non-PrEP users (1.09), with a statistically significant difference (*p* = 0.002). Similarly, the mean frequency of sex-related words was also greater among PrEP users (0.93) compared to non-PrEP users (0.53), with a significant difference (*p* = 0.01).

#### Location

**Fig. 8:**
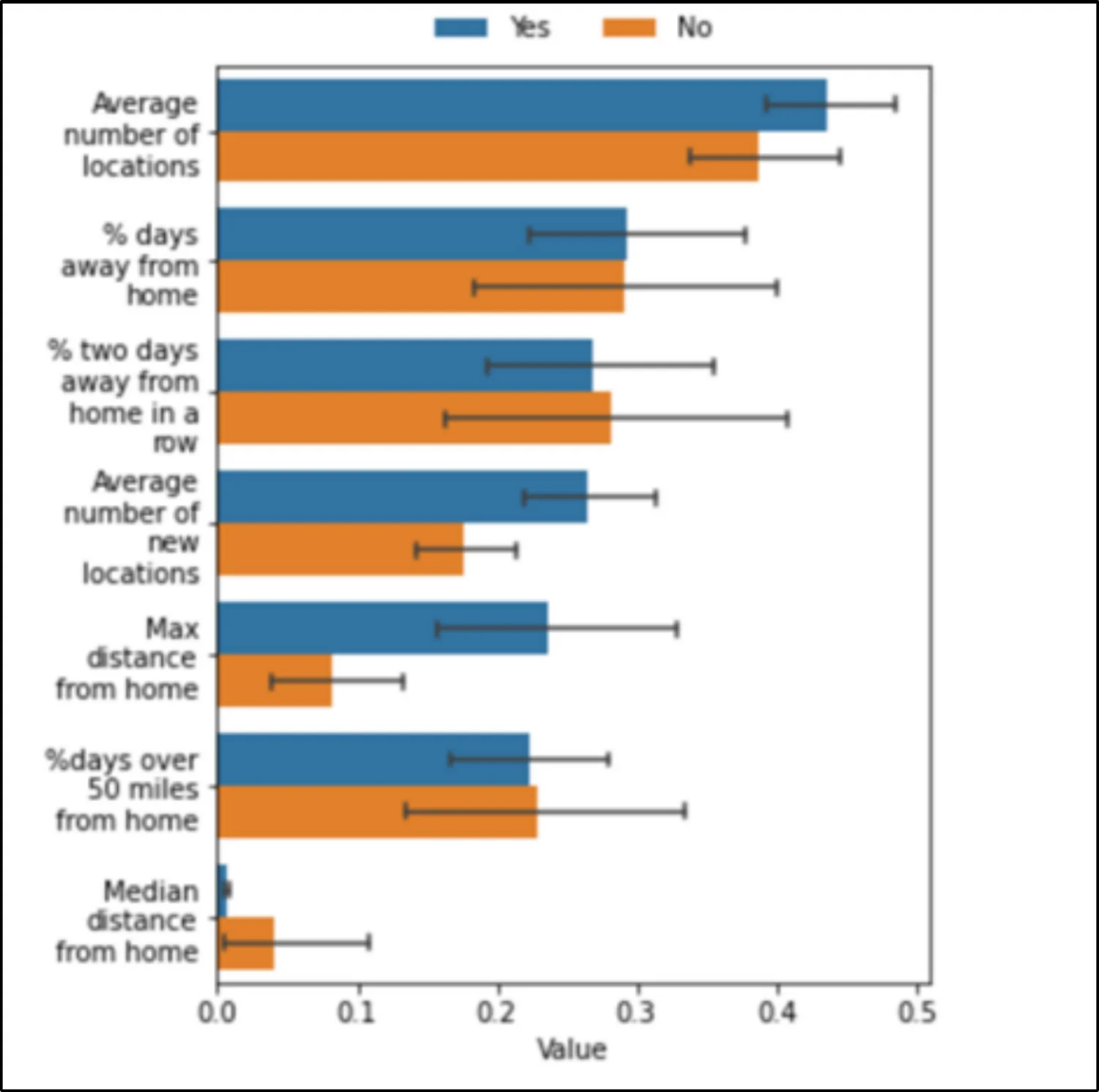
Normalized mean mobility measures for PrEP users and non-users, with error bars indicating standard deviations. PrEP users reported greater movement across several indicators, including average number of locations, distance from home, and new locations visited

Location data, shown in Fig. 8, revealed a significant difference in the normalized mean for the maximum distance traveled from home, with PrEP users traveling farther on average (0.24) compared to non-users (0.08, *p* = 0.007). Additionally, PrEP users visited a greater number of new locations (0.26) compared to non-users (0.18, *p* = 0.01), suggesting higher mobility among PrEP users. Other location-based features, including the percentage of days away from home (*p* = 0.97), the percentage of two consecutive days away from home (*p* = 0.86), the percentage of days over 50 miles from home (*p* = 0.39), the average number of locations visited (*p* = 0.19), and the median distance from home (*p* = 0.22), did not show statistically significant differences in the means between groups.

#### LIWC

**Fig. 9:**
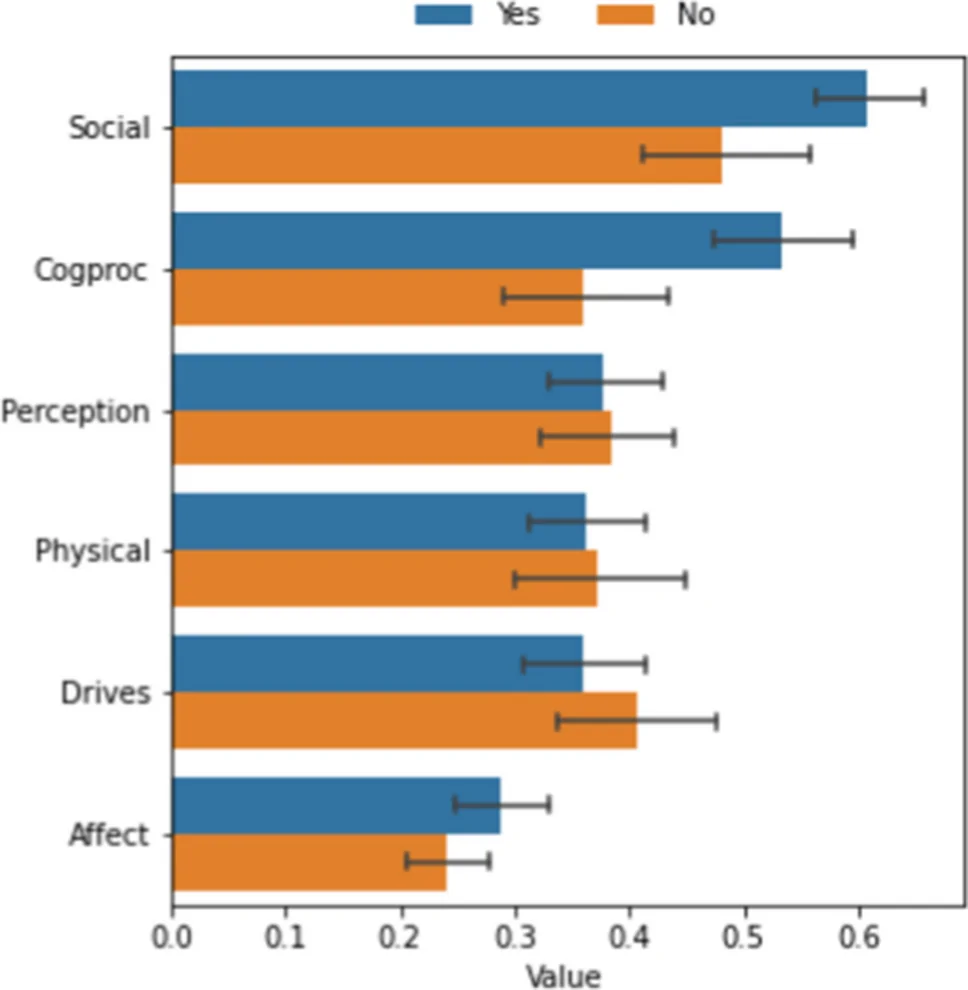
Normalized mean use of LIWC linguistic categories among PrEP users and non-users, with error bars showing standard deviations. PrEP users demonstrated higher average use of social, cognitive process, and affect-related words compared to non-users, while differences were smaller across other categories

LIWC analysis shown in Fig. 9 revealed significant differences in the normalized means of use of social and cognitive process words between PrEP users and non-users. PrEP users were significantly more likely to use social words (0.61) compared to non-users (0.48, *p* = 0.004) and cognitive process words (0.53) compared to non-users (0.36, *p* = 0.0005). These findings suggest that PrEP users engage in more social and analytical language. No significant differences were observed for perception words (*p* = 0.87), physical words (*p* = 0.85), drive-related words (*p* = 0.30), or affective words (*p* = 0.13).

## Discussion

Overall, our machine learning models demonstrated good predictive accuracy for both PrEP use and non-use, with F1 scores of 0.84 and 0.82, respectively. The models incorporating a combination of text input data collected from any app, including messaging, social media, and dating apps, were most predictive of PrEP use among the young SGM individuals in our study. This novel approach to predicting PrEP use status has several implications for HIV prevention research, which we discuss below.

In our study, PrEP users differed from non-users across multiple sociodemographic, behavioral, location, and text-based characteristics. PrEP users were found to be more active users of dating apps than PrEP non-users, which may indicate that SGM using these apps to find sexual partners were also more likely to use PrEP. This finding aligns with an earlier study which showed that Grindr users were more likely to take PrEP than SGM who did not use the app (Hoenigl et al., 2020). We also found that PrEP users reported a higher proportion of methamphetamine use and receptive condomless anal sex, and were more likely to use both sex- and drug-related words compared with participants who do not use PrEP. Substance use may be related to PrEP use because people who use substances are more likely to have more sexual partners (Cavazos-Rehg et al., 2011) and condomless anal sex (Boone et al., 2013), which, in the absence of PrEP, increases risk for HIV acquisition. In addition, injection drug use also increases risk for HIV; and therefore, injection drug users may be more likely to also use PrEP (Streed et al., 2022). However, relatively few participants in our study reported using injection drugs.

PrEP users traveled farther from home on average and visited more distinct locations. These mobility measures capture overall movement away from home, including routine commuting or other daily travel, and therefore should not be interpreted solely as discretionary or leisure travel. Frequent travel may both expand social networks associated with HIV risk and provide greater proximity to healthcare services, either of which could influence PrEP use, though we cannot disentangle these possibilities in the present study. Their LIWC normalized mean for cognitive processes was also higher, indicating more complex writing patterns (Tausczik & Pennebaker, 2010). This difference may partly relate to demographic factors such as age, as PrEP users were slightly older on average and younger adults are generally less likely to use PrEP relative to their risk level (Caponi et al., 2019; Snowden et al., 2017). Prior research shows that interest in PrEP uptake tends to decline at older ages (Klein and Washington, 2020; Hulstein et al., 2022), but subtle differences between early-20 s and late-20 s participants may still shape both mobility and language patterns.

Across all features and combinations, F1 score calculations were consistently better for the positive, or PrEP use outcome, rather than the negative, non-use outcome. When using all feature categories combined (Table 3, “All” row; apps + location + LIWC + risk-indicating words + BERT), the gradient boosting model yielded worse performance (F1 = 0.72 (PrEP use) and 0.59 (non-use)) than the best-performing All Apps model (F1 = 0.84 (PrEP use) and 0.82 (non-use)), indicating that adding every feature type did not improve accuracy. This suggests that the model trained on our sample may be less reliable in identifying participants who were not using PrEP, potentially indicating that the data contained stronger patterns for participants who were using PrEP.

To clarify how the models generated these predictions, we examined feature importance for both gradient boosting and logistic regression. Gradient boosting highlighted Grindr use as the most influential predictor of PrEP status, followed by other app engagement metrics such as xHamster, Google Home/Maps, and WhatsApp Messenger. Logistic regression coefficients likewise identified Grindr and overall messaging activity as positively associated with PrEP use, while certain location and language features were negatively associated (Figs. 2 and 3). These interpretability analyses demonstrate which specific digital behaviors drive the model’s predictions and provide actionable insights for future PrEP promotion efforts.

Accurate prediction of PrEP use and non-use can guide the design of targeted PrEP uptake interventions for young SGM at highest risk for HIV. Grounded in the Information–Motivation–Behavioral Skills framework (Fisher & Fisher, 1992; Fisher et al., 2003), this approach focuses on the key drivers of PrEP initiation: providing accurate information, strengthening motivation, and building the skills needed to start and sustain PrEP. By detecting individuals not currently on PrEP, passively collected mobile data could trigger timely outreach that delivers tailored education and links participants to PrEP providers or other sexual health services. Just-in-time notifications might include motivational messages, information on nearby clinics or other sexual health resources, or guidance for navigating insurance and cost concerns. For those already on PrEP, the same system could reinforce adherence through refill reminders, medication prompts, or testing support. At the population level, aggregated de-identified data could track PrEP coverage and highlight geographic or demographic gaps, enabling public health agencies to allocate HIV prevention resources where motivation and behavioral supports are most needed. In sum, this proof-of-concept study highlights the need for next-step validation in larger, more diverse cohorts and exploration of scalable applications across platforms to strengthen its contribution to HIV prevention research.

Though our models were most predictive of PrEP use status, they were not perfect, meaning that PrEP use status was misidentified for some individuals. This could have serious consequences. Misidentifying a PrEP non-user as a PrEP user and vice versa would result in inappropriate and irrelevant notifications, and in some cases, a missed opportunity for care for individuals at risk for HIV. As this was a relatively smaller sample used to train our model, some misclassification was expected, but future studies with validation self-report data should investigate whether the models are biased to misclassify a particular demographic group more than others, which could inadvertently perpetuate disparities in PrEP access and adherence. Because these predictions involve sensitive health behaviors, strong safeguards for privacy and data security are essential. Any deployment of such models should ensure robust encryption, limited data retention, and clear informed consent so that participants understand how their data will be used and can opt out at any time. Ethical oversight will be critical to prevent unintended disclosure or misuse of personal information and to maintain community trust.

While the potential benefits of passive data collection are numerous, ethical and privacy concerns must be considered. Ensuring informed consent for data collection is crucial. Users should have the ability to “opt in” to data-driven PrEP notifications, with access to information on the type of data that is being collected and how their data will be used. As employed in the current study, participants had the flexibility to turn off the data collection at any time, ensuring participant autonomy. Confidentiality should be prioritized through robust data protection measures and compliance with HIPAA and other relevant regulations. Community involvement and oversight in the use of ML to predict PrEP use are also essential for its ethical implementation. In the current study, semi-structured interviews facilitated the refinement of the sexual risk and substance use keywords. Though the “risk-indicating words” feature yielded worse predictive accuracy, we believe that the process of direct community engagement in feature development highlights an important and replicable practice that enhances model relevance for diverse populations. Finally, although we adapted our library of risk-indicating words from previously published, community-validated work (Ovalle et al., 2021) and refined it with participant feedback, we did not directly validate each term against observed sexual or substance use behavior within this dataset. Our use of this feature should therefore be interpreted as a proof-of-concept measure aligned with the Information–Motivation–Behavioral Skills framework (Fisher & Fisher, 1992; Fisher et al., 2003) rather than a confirmed behavioral indicator.

Our study has several limitations, in addition to the risks from misidentification of PrEP use status, that should be considered when interpreting our findings. First, our study was limited to Android users, making it non-generalizable to iOS users. Because mobile use patterns and demographic characteristics can differ between Android and iOS users (e.g., age, income, regional distribution), the behaviors captured in our passive data may not fully reflect those of the broader SGM population. Future research in this area ideally should include iOS users or employ cross-platform tools to ensure findings are representative and to evaluate whether operating-system differences affect model performance. Of 103 participants enrolled in the study, 41% reported PrEP use at baseline, whereas of the 82 participants in our analytical sample, 56% reported PrEP use at baseline. This suggests that participants who did not report PrEP use were less likely to provide the 30 days of data necessary for inclusion in the current study, indicative of potential selection bias. Certain sociodemographic questions were added at a later time, resulting in missingness across several covariates in Table 1. Thus, investigating differences in sociodemographic characteristics between those included/excluded from the study poses a challenge. While most sociodemographic and behavioral variables did not differ between participants included in the analytic sample and those excluded, PrEP use was significantly higher among included participants. This difference raises the possibility of selection bias related to PrEP use, and our findings should be interpreted with that context in mind. Lastly, the passive data collection was from a singular Android device for each participant, which does not account for common use of multiple devices (i.e., laptop, desktop computer, tablet).

## Conclusions

This study shows the promise of integrating mobile app-collected data and ML into HIV prevention efforts, particularly for SGM young adults. Using keystroke input, app use metrics, and location data collected from the study’s eWellness app, we assess a variety of features and their ability to predict PrEP use status and explore differences in behavioral patterns between PrEP users and non-users. These findings suggest that passively sensed data could trigger just-in-time, theory-driven interventions that provide tailored PrEP education, motivational messaging, and support for overcoming structural barriers. Future research should validate and refine these predictive models across broader populations and test their capacity to deliver scalable, privacy-protective interventions, while maintaining strong community involvement and strict data security and ethical safeguards.

## Data Availability

While the datasets are restricted from public release to protect participant privacy, interested researchers can request access from the corresponding author, who will outline conditions for responsible data sharing.
